# Exogenous Methyl Jasmonate Alleviates Mechanical Damage in Banana Fruit by Regulating Membrane Lipid Metabolism

**DOI:** 10.3390/foods13193132

**Published:** 2024-09-30

**Authors:** Chunxia Huang, Ping Yi, Jing Li, Lihong Xie, Fang Huang, Min Huang, Ting Gan, Jian Sun, Li Li

**Affiliations:** 1 College of Chemistry and Bioengineering, Guilin University of Technology, Guilin 541006, China; hchunxia202203@163.com (C.H.); ruochenjl@163.com (J.L.); 2 Agro-Food Science and Technology Research Institute, Guangxi Academy of Agricultural Sciences, Nanning 530007, China; pingyi@gxaas.net (P.Y.); lihong@gxaas.net (L.X.); huangfang@gxaas.net (F.H.); hmin@gxaas.net (M.H.); ganting@gxaas.net (T.G.); 3 Guangxi Key Laboratory of Fruits and Vegetables Storage-Processing Technology, Guangxi Academy of Agricultural Sciences, Nanning 530007, China; jiansun@gxaas.net; 4 Guangxi Academy of Agricultural Sciences, Nanning 530007, China

**Keywords:** banana fruit, methyl jasmonate, mechanical damage, membrane lipids, enzyme activities

## Abstract

Bananas are economically important fruits, but they are vulnerable to mechanical damage during harvesting and transport. This study examined the effects of methyl jasmonate (MeJA) on the cell membrane integrity and membrane lipid metabolism of wounded banana fruits after harvest. The results showed that 10 and 50 μM MeJA treatments on mechanically wounded bananas significantly delayed ripening and senescence in comparison with the control. At the end of storage, MeJA-treated groups showed a significant reduction in electrolyte leakage and malondialdehyde content, indicating that MeJA protected cell membrane integrity. MeJA also led to a significant decrease in the activity of antioxidant enzymes, including lipoxygenase, diacylglycerol kinase, and lipid phosphate phosphatase. Furthermore, MeJA reduced phospholipase (C and D), phosphatidic acid, and diacylglycerol levels, as well as slowed down the decrease in phosphatidylcholine and phosphatidylinositol contents. Compared to the control, MeJA significantly downregulated the expression of *MaPLDγ*, *MaPLDα*, and *MaPLDζ*. Therefore, MeJA treatment could be a reliable method to delay the senescence of harvested banana fruits subjected to mechanical wounding.

## 1. Introduction

Bananas (*Musa acuminata* L.) are attractive, delicious, and nutritious tropical/subtropical fruits. They are popular worldwide and play an important role in commercial exchange [1,2,3]. However, they exhibit typical climacteric behavior, i.e., their physiological metabolism is very active, and their disease resistance decreases after harvest [4,5]. During storage and transportation, bananas are susceptible to mechanical damage, which results in deteriorating tissue structure and increasing sensitivity to pathogen invasion [6]. These factors hasten fruit ripening and rotting, thereby reducing banana fruit quality and inducing significant economic loss [5].

Previous studies have indicated that aging, softening, and browning are significantly influenced by membrane lipid metabolism in fruits and vegetables [7,8]. Membrane deterioration is an early and important characteristic of plant cells that are mechanically damaged. When plants are damaged, each wounded cell stimulates other cells to activate a defense response. For example, phospholipase D (PLD) has been found to be activated in postharvest bananas [9] and cucumbers [10] following mechanical damage, which is involved in the signaling pathways responding to mechanical stress by upregulating the expression of *PLD* genes and exacerbating cell membrane damage. In addition, mechanical damage increased the accumulation of endogenous jasmonic acid (JA) in *Arabidopsis*, which activated wound response and JA response genes [11]. The local release of JA at the wound site systematically enhanced the resistance of distant parts in the plant and played a role as a long-distance signal [12]. The biosynthesis of JA through octadecanoid pathway began with the release of linolenic acid from membrane and induced the production of various volatiles, which significantly accumulated following plant damage [13].

Among plant volatiles, methyl jasmonate (MeJA) is a plant hormone that belongs to the JA family. MeJA serves as a signaling molecule during stress reactions in plants responding to environmental changes and infections [14]. This function extends to its broad application potential in regulating postharvest quality, enhancing chilling resistance, improving antioxidant systems, and promoting the accumulation of nutritional compounds in fruits and vegetables, such as okra [15], mango [16], grape [17], and broccoli [18]. Exogenous MeJA stimulates the production of defensive compounds in plants and triggers the expression of genes linked to both local and systemic acquired resistance. This results in the accumulation of active compounds and the promotion of stressed plant growth. Moreover, it changes endogenous hormone levels and other physiological and biochemical properties of plants [15,19]. Lipoxygenase (LOX) plays an important role in the production of MeJA by promoting the conversion of linoleic and linolenic acids into peroxides [6,12]. Phospholipases play an important role in phospholipid metabolism through catalyzing the hydrolyses of phosphatidylinositol (PI), phosphatidylcholine (PC), and other phospholipids to produce signaling molecules, such as diacylglycerol (DAG) and phosphatidic acid (PA), which are involved in signal transduction [5]. Lipid phosphate phosphatase (LPP) is an essential component of lipid metabolism and signal transmission because it facilitates PA dephosphorylation to produce DAG. Diacylglycerol kinase (DGK) generates PA, which is involved in membrane lipid metabolism by phosphorylating DAG [6]. Exogenous MeJA controls the enzyme activities of LOX, LPP, PLC, PLD, and DGK, which in turn affects amount and metabolism of PA, DAG, and other membrane lipid components. This regulates the metabolism and signal transduction of membrane lipids, slows down membrane degradation, and preserves the structural integrity of membranes [20,21].

MeJA has been widely used to improve the stress resistance and storage quality of fruits and vegetables. However, there are few reports on how exogenous MeJA assists plants in resisting external threats causing physical damage. Therefore, in the present study, bananas were used to examine how plants resist damage during exogenous MeJA treatment when subjected to local injury. Bananas were treated with varying concentrations of MeJA to determine the effects of exogenous MeJA on mechanical injury. The effects of different MeJA concentrations on the activities of membrane lipid metabolism-related enzymes and phospholipid content were measured in banana peels that were damaged after harvest. In addition, quantitative real-time PCR (qRT-PCR) was used to measure *MaPLD* expression in mechanically injured banana peels following MeJA treatment to determine its effect on banana membrane lipid metabolism.

## 2. Materials and Methods

### 2.1. Plant Materials and Treatment

Banana fruits (*Musa acuminate* AAA cv. ‘Guijiao No. 1’) were harvested at mature green stage (firmness: 378.86 N; total soluble solid content: 3.53; hue angle value: 109.87) from an orchard located in Tanluo town of Nanning city, Guangxi province, China (22°55′21.7″ N, 107°58′26.9″ E). They were immediately transported to a laboratory within 2 h. Fruits exhibiting uniform ripeness, size, shape, mechanical damage, or pest infestation were selected out. These fruits were rinsed and disinfected with 0.1% sodium hypochlorite solution for 10 min. To simulate mechanical damage, a stainless-steel puncher was used to create three wounds that were 3 cm apart at a depth of 2 mm and a diameter of 4 mm on peels [9]. The MeJA (Macklin Biochemical Co., Ltd., Shanghai, China) solutions were prepared by dissolving MeJA with a purity of 95% in distilled water to achieve concentrations of 10 μM and 50 μM. Then, fruits were randomly divided into three groups and soaked in distilled water (Control), 10 μM MeJA, or 50 μM MeJA solutions for 10 min. The fruits were packaged in unsealed polyethylene film bags (0.03-mm thick) and stored at 25 °C and 90–95% relative humidity in the dark for 15 days. During storage, peel tissues were collected on days 0, 3, 6, 9, 12, and 15 to evaluate the effects of MeJA at different time points. Samples were lyophilized in liquid nitrogen and stored at −80 °C until further analysis.

### 2.2. Analyses on Electrolyte Leakage and Malondialdehyde Content

Electrolyte leakage was determined using 10 discs of banana peel tissues according to the method of Li et al. [22]. Malondialdehyde (MDA) content was measured as described previously by Lin et al. [23] with 1 g of peel tissue and expressed as nmol g^−1^ fresh weight (FW).

### 2.3. Assay of LOX, LPP, and DGK Activities

Enzyme activities were measured according to the method of Li et al. [9], with slight modifications. Banana peels (0.1 g) were added to 0.9 mL of pre-cooled phosphate buffer (pH 7.8) and thoroughly shaken. The solution was centrifuged at 3500× *g* for 15 min at 4 °C, and the supernatant was collected. LOX, LPP, and DGK activities were measured using ELISA kits (Jiangsu Meimian Industrial Co., Yancheng, China). OD values were measured at 450 nm, and enzyme activities were determined based on standard curves prepared from the OD values of a set of standards. LOX, LPP, and DGK activities were expressed as U/g of FW.

### 2.4. Determination of PLC and PLD Contents

PLC and PLD contents were measured using ELISA kits (Jiangsu Meimian Industrial Co., Yancheng, China) according to the methods described by Li et al. [6], with some modifications. The sample solution was obtained in the same way as described in Section 2.3. OD values were measured at 450 nm and enzyme contents were determined based on standard curves prepared from the OD values of a set of standards. PLC and PLD levels were displayed as μg/g FW of banana peel.

### 2.5. Measurement of Cell Membrane Phospholipid Contents

PI, PA, PC, and DAG contents were measured following the procedure described by Chen et al. [24] with some modifications. Banana peel powder (0.1 g) was homogenized in 1 mL of methanol solution (2:1, *v*/*v*). The homogenate was centrifuged at 10,000× *g* for 20 min and extracted for 30 min via ultrasonication. After adding acetone and chloroform, the homogenate was dried under nitrogen stream and redissolved in Folch solution. The mixture was passed through a 0.22 μm filter. The amount of membrane phospholipid was expressed as μg/g FW of banana peel.

### 2.6. Analysis on PLD Gene Expression in Cell Membranes

The total RNA from banana peels was extracted using a RNAprep Pure Plant Kit (TIANGEN, Beijing, China) based on the manufacturer’s instructions. Complementary DNA (cDNA) from banana peels was synthesized using the Hiscript^®^ II RT SuperMix kit (Vazyme, Nanjing, China) following the manufacturer’s protocol. The expression of three *PLD* genes in banana (*MaPLDγ*, *MaPLDα*, and *MaPLDζ*) were analyzed using the method described by Yi et al. [25], and the banana CAC gene was selected as the internal reference gene.

### 2.7. Data Analysis

Each experiment was performed three times. All data were presented as mean value ± standard error. Experimental data were analyzed using one-way analysis of variance on SPSS 26.0 software (IBM Corp., Armonk, NY, USA). Mean differences were determined using Fisher’s least significant difference test (*p* < 0.05).

## 3. Results

### 3.1. Electrolyte Leakage and MDA Content

**Figure 1:**
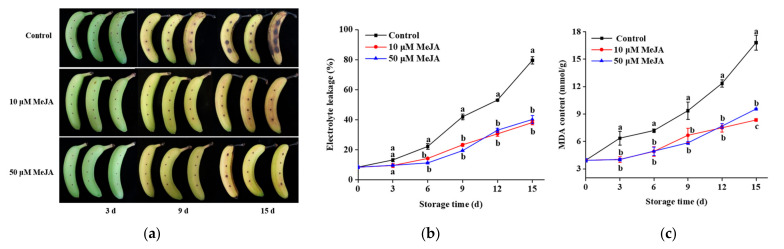
Effects of different treatments on banana appearance (**a**), cell electrolyte leakage (**b**), and MDA content (**c**) during storage. Significant differences (*p* < 0.05) among different treatments on the same storage day were indicated by different lowercase letters.

As shown in the Figure 1a, on day 9, the banana peels had turned yellow, and the mechanical damage was noticeably more severe in the control group compared to the MeJA-treated groups. On day 15, this difference became even more pronounced, with the control group showing greater mechanical damage and signs of aging compared to the MeJA-treated bananas. The integrity of the cell membrane in the fruit peels was disrupted by mechanical damage, resulting in the leakage of cellular electrolytes and consequent alterations in membrane conductivity, which serve as indicators of changes in ion permeability across cell membrane [21]. As seen in Figure 1b, the electrolyte leakage of the banana peels gradually increased throughout storage. Throughout the storage period, a slower increase in electrolyte leakage occurred in the 10 and 50 μM MeJA groups, with significant differences observed from day 6 compared to those in the control group. However, no significant difference in electrolyte leakage was evident between the 10 and 50 μM MeJA groups. The reduction in electrolyte leakage indicated the effectiveness of the MeJA treatment in maintaining membrane integrity, which helped mitigate the effects of mechanical damage on the fruit peels. By reducing electrolyte leakage, the MeJA treatment likely delayed the membrane degradation process, thereby slowing down the deterioration of fruit quality. This effect was also reflected in the appearance of the bananas.

The formation of MDA results from the peroxidation of membrane lipids during banana storage and through a reaction with proteins and nucleic acids. This results in alterations in molecular configuration and disruption to cell membrane integrity. Therefore, the MDA content indicates the membrane lipid oxidation degree, which also reflects the extent of fruit aging and damage during storage [13]. An increasing trend in the MDA content was observed in the banana peels during storage (Figure 1c). Banana peels treated with 10 and 50 μM MeJA exhibited a steady increase in MDA content throughout the storage period, which was lower than control. On day 15, the MDA contents for the 10 and 50 μM MeJA groups were 9.54 and 8.33 mmol/g, which were 50.4% and 43.2% lower than the control group. The reduction in MDA content indicated that the MeJA treatment slowed down the membrane lipid oxidation process, thereby reducing membrane damage. This was related to the inhibition of LOX activity, as LOX was involved in the oxidation of membrane lipids. By reducing MDA formation, the MeJA treatment helped maintain membrane integrity and improve fruit storage quality. As shown in the Figure 1a, on day 15, the reduction in MDA content corresponded with the visual differences in fruit appearance. The control group exhibited more mechanical damage and signs of aging, while the MeJA-treated bananas showed better peel integrity and less mechanical damage.

### 3.2. LOX, LPP, and DGK Activities

**Figure 2:**
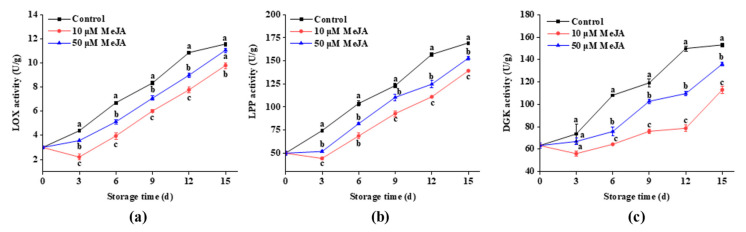
Effects of 10 and 50 μM MeJA treatments on LOX (**a**), LPP (**b**), and DGK (**c**) activities in bananas during storage. Significant differences (*p* < 0.05) among different treatments on the same storage day were indicated by different lowercase letters.

LOX is widely distributed in plants and animals. It is a naturally occurring protein involved in lipid breakdown, the regulation of growth and development, aging, and defense response [26]. From Figure 2a, throughout the storage period, a general upward trend in LOX activity in banana peels was observed. On day 3, there was a marked increase in LOX activity in control and 50 μM MeJA-treated groups; however, 10 μM MeJA group showed a decreasing trend in LOX activity for the first 3 days, followed by a significant rise. In contrast to the 10 and 50 μM MeJA groups, the control group exhibited significantly higher LOX activity on days 3, 6, 9, and 12 during the storage period. Additionally, the 10 μM MeJA group maintained a considerably lower level of LOX activity compared with the 50 μM MeJA group. At the end of storage, the 10 μM MeJA group had a LOX activity of 9.8 U/g, which was 11.3% and 15.2% lower than the 50 μM MeJA and control groups. The reductions in LOX activity directly contributed to the inhibition of lipid peroxidation, resulting in better membrane integrity and reduced mechanical damage in banana peels, as lower LOX activity reduced the breakdown of membrane lipids.

LPP is a phosphatase enzyme found on cell membranes and is involved in signal transduction [27]. From Figure 2b, an increasing trend in LPP activity was observed in banana peels, with significantly higher LPP activity in the control group compared to the 10 and 50 μM MeJA-treated groups throughout the storage period. In contrast to the control group, the 10 and 50 μM MeJA groups showed marked changes in LPP activity, which initially decreased during the first 3 days before significantly increasing. The LPP activities at the end of storage were 139.14 and 152.78 U/g in the 10 and 50 μM MeJA groups, which were 17.8% and 9.7% lower than the control group. These results indicate that the 10 and 50 μM MeJA groups had a stronger inhibitory effect on LPP activity, suggesting that MeJA might be involved in the regulation of the signaling pathways associated with phospholipid metabolism.

DGK is an enzyme that catalyzes the phosphorylation of DAG to PA and plays an important role in the growth, development, and adaptation processes of plants. It is also involved in plant lipid signaling [28]. From Figure 2c, an increasing trend in DGK activity was observed in the banana peels during the storage period. For the first 3 days, no significant difference was observed in the DGK activity among the three groups, whereas a significant difference was observed on days 6, 9, 12, and 15. On day 15, DGK activities for the 10 and 50 μM MeJA groups were 26.2% and 11.2%, which were lower than the control group.

These results collectively demonstrate that the inhibition of LOX, LPP, and DGK activities by the MeJA treatment played a crucial role in maintaining membrane integrity, reducing lipid peroxidation, and enhancing the resistance of banana peels to mechanical damage during storage.

### 3.3. PLC and PLD Contents

**Figure 3:**
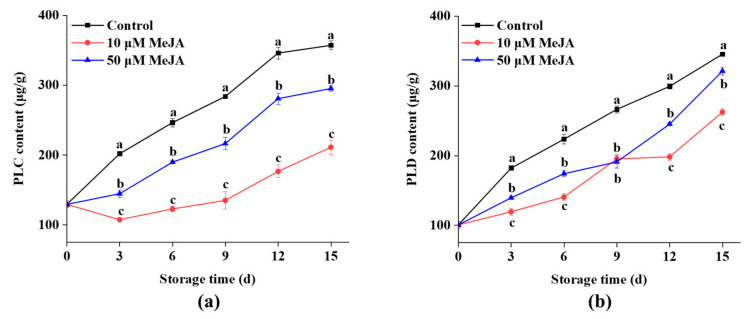
Effects of 10 and 50 μM MeJA treatments on PLC (**a**) and PLD (**b**) contents in bananas during storage. Significant differences (*p* < 0.05) among different treatments on the same storage day were indicated by different lowercase letters.

The PLC enzyme hydrolyzes cell membrane phospholipids into DAG and inositol trisphosphate (IP3), which are essential for plant development, growth, and stress response [29]. Figure 3a showed that the PLC content in the banana peels steadily increased throughout the storage period, with significant differences between groups. The PLC content quickly increased after day 3 in both the control and the 50 μM MeJA groups, then it gradually leveled off on days 12 and 15. Compared to the control and the 50 μM MeJA groups, the PLC content in the 10 μM MeJA group gradually increased during storage. On day 15, the 10 μM MeJA group exhibited a significantly lower PLC content, which was 40.9% and 17.3% lower than the control and 50 μM MeJA groups.

PLD is an important transmembrane signal transduction enzyme that primarily converts PC into PA and free choline. This affects membrane structure, function, and stability, and it plays an important role in signal transduction, defense response, and other physiological processes [30]. As seen in Figure 3b, there was a continuous increase in the PLD content in the banana peels throughout the storage period. The PLD content in control group sharply increased from day 0 onwards, and this trend persisted throughout the storage period. It was significantly higher than that in the 10 and 50 μM MeJA groups. On day 15, the PLD levels in the banana peels from the 10 and 50 μM MeJA groups were 262.40 and 321.17 μg/g, which were 24.0% and 7.0% lower than the control group.

### 3.4. Membrane Phospholipid Content

**Figure 4:**
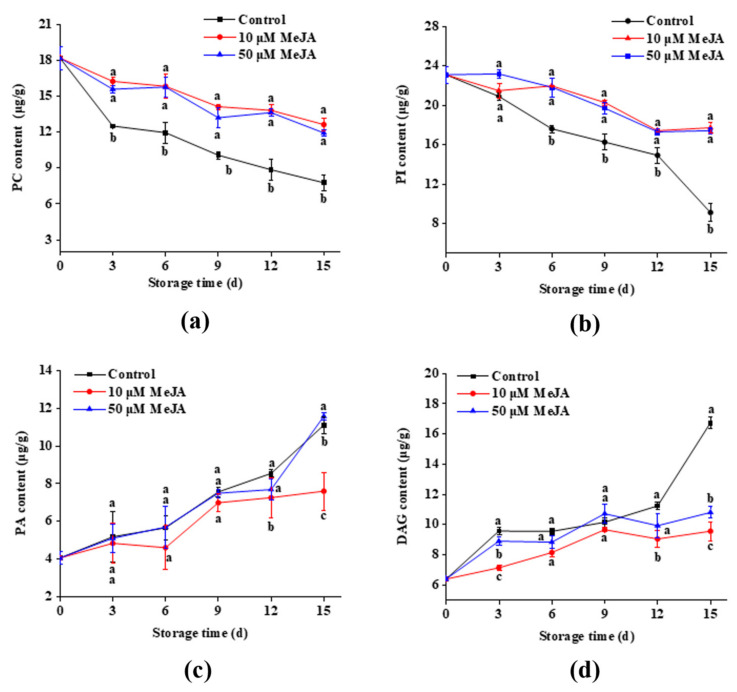
Effects of 10 and 50 μM MeJA treatments on PC (**a**), PI (**b**), PA (**c**), and DAG (**d**) contents in bananas during storage. Significant differences (*p* < 0.05) among different treatments on the same storage day were indicated by different lowercase letters.

Phospholipids are the major constituents of plant cell membranes, and the composition of phospholipids in cell membranes is complex, including PA, PC, DAG, and PI. These phospholipids collectively form the structural foundation of cell membranes and engage in various physiological activities that occur within the cell, including signal transmission, membrane repair, and lipid metabolism [31]. As seen in Figure 4, the PC and PI in the banana peel cell membranes were hydrolyzed during mechanical stress, which resulted in the accumulation of compounds such as PA and DAG with an increase during storage. Consequently, there was a decrease in the quality of fruits and vegetables during postharvest storage because of an increase in membrane permeability.

Figure 4a shows that the control group had a significant decrease in its PC content during storage, whereas the 10 μM MeJA group exhibited a gradual decrease. However, the 50 μM MeJA group showed a decreasing trend with fluctuation. Throughout the storage period, the PC content in the control group was significantly higher than that in the 10 and 50 μM MeJA groups. However, there was no significant difference in the PC content between the two MeJA treatment dosages. Figure 4b shows that the bananas in the control group had a significant drop in PI content during storage; however, the 10 and 50 μM MeJA groups showed a gradually decreasing trend. From day 6, the PI concentration in the control group was significantly increased compared to those concentrations in the 10 and 50 μM MeJA groups. Nevertheless, throughout the storage period, no discernible variation in PI content was observed across the various MeJA treatment concentrations. On the last day of storage, the PC content in banana peels from the control groups was 38.5% and 32.8% lower than that in peels from the 10 and 50 μM MeJA groups, whereas the PI content was 48.5% and 47.7% lower than that in peels from the 10 and 50 μM MeJA groups.

The PA content in the 50 μM MeJA and control groups increased significantly between days 6 and 15 (Figure 4c). After a modest reduction on days 3 and 6, the PA content in the 10 μM MeJA group slowly increased on days 9, 12, and 15. The PA content in the 10 μM MeJA group was 31.5% and 31.4% lower than the 50 μM MeJA and control groups on day 15. The DAG content in the 10 and 50 μM MeJA groups increased at first, then somewhat decreased, and increased once again (Figure 4d). Moreover, the DAG content in the control group was considerably higher than the 10 and 50 μM MeJA groups on days 3 and 15. On day 15, the DAG concentration for the 10 and 50 μM MeJA groups were 9.56 and 10.80 μg/g, which were 42.8% and 35.4% lower than the control group.

These results showed that the control group had a significant decrease in PC and PI content throughout the storage period, indicating pronounced phospholipid degradation and consequent membrane instability, which accelerated the deterioration of fruit quality. In contrast, the 10 μM and 50 μM MeJA groups exhibited significantly higher levels of PC and PI compared to the control group, suggesting that MeJA exerted a protective effect by slowing the degradation of membrane phospholipids. In the 10 μM MeJA group, the rates of increase in the PA and DAG content were slower compared to both the control and 50 μM MeJA groups, indicating that MeJA treatment mitigated phospholipid hydrolysis. Consequently, the lower accumulations of PA and DAG reflected a delay in membrane lipid breakdown and mechanical damage response.

### 3.5. Relative Expression of MaPLDγ, MaPLDα, and MaPLDζ

**Figure 5:**
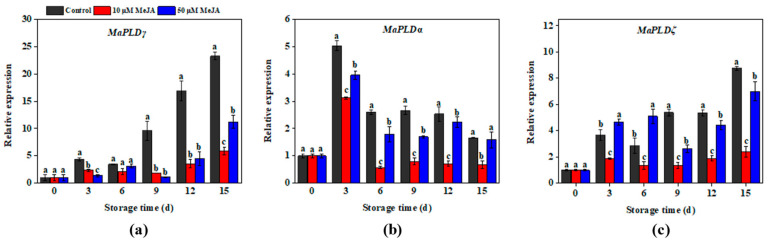
Effects of 10 and 50 μM MeJA treatment on the relative expression of *MaPLDγ* (**a**), *MaPLDα* (**b**), and *MaPLDζ* (**c**) in bananas during storage. Significant differences (*p* < 0.05) among different treatments on the same storage day were indicated by different lowercase letters.

To determine the effect of MeJA on *MaPLD* genes expression in mechanically damaged banana peels, the relative expression of *MaPLDγ*, *MaPLDα*, and *MaPLD*ζ were measured using qRT-PCR. The relative expression of *MaPLDγ* in banana peels showed an initial increase, followed by a decrease, and then another increase trend (Figure 5a). Except for days 0 and 6, the relative expression of *MaPLDγ* in control group was considerably higher than the 10 and 50 μM MeJA groups. On day 15, the expression levels of *MaPLDγ* in the 10 and 50 μM MeJA groups were 5.9 and 11.2, which were 74.4% and 51.7% lower than the control group. Similarly, Figure 5b shows an increasing trend, which subsequently turned into a decreasing trend, whereas the relative expression of *MaPLDα* in the banana peels peaked on day 3. There were significant differences in the relative expression of *MaPLDα* among the three groups on days 3, 6, 9, and 12. In addition, there were significantly lower expression levels between the 10 and 50 μM MeJA groups, which were 38.0% and 21.3% lower than the control group on day 3. In Figure 5c, the relative expression of *MaPLDζ* in the banana peels exhibited the same trend as *MaPLDγ*. On day 15, the expression levels of *MaPLDζ* in the 10 and 50 μM MeJA groups were 2.4 and 7.0, which were 72.3% and 19.8% lower than control group.

These results show that MeJA treatment significantly reduced the expression of *MaPLDγ*, *MaPLDα*, and *MaPLDζ*, inhibiting membrane phospholipid degradation and improving the stress resistance of mechanically damaged banana peels. These results support MeJA as an effective treatment method for enhancing fruit quality retention during postharvest storage.

## 4. Discussion

### 4.1. The Effect of Methyl Jasmonate on MDA Content

Cell membranes are essential for preserving the specialized structure of cells and enabling organisms to engage in regular physiological metabolic processes. Bananas are vulnerable to mechanical harm, such as collisions and squeezing during harvest and transport, which can destroy tissue structure in fruits [2]. Electrolyte leakage and MDA concentration are important factors for evaluating membrane integrity and lipid peroxidation. A damaged cell membrane can lead to cytoplasmic leakage, metabolic disruption, lipid peroxidation, and other physiological changes that accelerate ripening, aging, and keep the quality of longan fruit [32]. In the present study, we examined the effects of mechanical damage on electrolyte leakage and MDA content in banana peels. The increase in electrolyte leakage for banana peels was linked to prolonged storage, which promoted membrane lipid peroxidation caused by the loss of membrane integrity following stress from mechanical injury. Figure 1a shows that on day 9, the control group had visible mechanical damage compared to the MeJA-treated bananas, with the difference becoming even more pronounced on day 15. MeJA treatment significantly reduced the electrolyte leakage and MDA content in banana peels; however, there was no variation between the 10 and 50 μM MeJA groups during the storage period.

The delay in ripening caused by MeJA in bananas was related to its regulation of membrane lipid metabolism, which helped maintain membrane integrity and slow down the ripening process induced by mechanical damage. In contrast, the accelerated ripening effect of MeJA in tomatoes was associated with its mechanism of promoting ethylene production and the expression of related genes [33]. In strawberry, MeJA accelerated ripening by enhancing the expression of genes involved in pigment, sugar metabolism, fruit ripening, softening, and hormone synthesis pathways [34]. Similarly, MeJA regulates the accumulation of anthocyanins, volatile compounds, and chlorophyll, contributing to the development and ripening process of peach fruits [35]. This dual effect highlighted the complexity of MeJA’s mechanisms in different fruits and suggested that the type of fruit and specific treatment conditions needed to be considered when applying MeJA. Our results indicate that the MDA content and electrolyte leakage in banana fruits following mechanical stress were not significantly affected by MeJA concentration beyond a certain limit. Moreover, other reports have suggested that MeJA treatment significantly decreases electrolyte leakage and MDA content accumulation [20,36,37]. The results of this study indicate that MeJA treatment effectively maintained membrane integrity and protected banana fruit quality.

### 4.2. The Effect of Methyl Jasmonate on LOX, LPP, and DGK Activities

The breakdown of membrane lipids occurs during the loss of cell membrane integrity in plants. Increased activities of membrane lipid metabolic enzymes (e.g., PLD, LOX, LPP, and DGK) were the primary cause of membrane lipid breakdown [38]. Enzymes linked to the breakdown of membrane lipids were triggered by mechanical stress, which resulted in the hydrolysis of phospholipids on the cell membrane, including PC and PI. The products of hydrolysis, such as PA and DAG, function as signaling molecules that were involved in several physiological processes, such as aging and cellular defense. Therefore, controlling the activity of enzymes involved in membrane lipid metabolism and the suppression of membrane lipid breakdown can enhance defense against external environmental stress and even achieve damage repair in plants. In the present study, the 10 and 50 μM MeJA groups exhibited a significant reduction in LOX, LPP, and DGK activities during storage, and they decreased the PLC and PLD levels compared to the control group. Moreover, the results indicated that the 10 μM MeJA group showed a more pronounced effect. It was noteworthy that during the early stages of storage, the 10 μM MeJA group exhibited a decreasing trend in LOX, LPP, and DGK activities, as well as PLC content. This early decrease might have reflected that MeJA, in the initial phase, inhibited the activity of these enzymes to slow down membrane lipid degradation, thereby protecting the cell membrane from mechanical damage and early oxidative stress. Although lower enzyme activities and PLC content might have indicated reduced membrane lipid degradation, the short-term inhibitory effect of MeJA helped maintain membrane stability in the early stage. Shuai et al. observed that in longan fruit treated with MeJA, the LOX activity initially decreased slightly and then increased during the early stages of storage [21]. In contrast, the 50 μM MeJA group did not show a similar early decline, which might have been related to its higher concentration. Different concentrations of MeJA might have had varying regulatory effects on membrane lipid metabolic enzymes. The 10 μM MeJA might have been more effective in inhibiting enzyme activity in the early stage, whereas the 50 μM MeJA could have influenced different regulatory mechanisms due to its higher concentration or exhibited different regulatory characteristics at the early stage. Furthermore, some studies indicated that MeJA affected the metabolism of membrane lipids in ‘Hass’ avocados and peaches, and it delayed the activation of genes associated with membrane lipid metabolism and phospholipid breakdown [39,40]. The present results also indicated that membrane lipid peroxidation was delayed by MeJA, LOX activity was inhibited, LPP and DGK activities were suppressed, and phospholipase content was reduced, thereby inhibiting phospholipid degradation in cell membranes of banana fruits. This contributed to the stability maintenance of membrane system and the enhancement of postharvest tolerance in fruits.

### 4.3. The Effect of Methyl Jasmonate on Phospholipase

The present study revealed that mechanical damage could stimulate plants to produce defense signals that were involved in stress defense to counter environmental stress [41,42]. In addition, PLD is an essential enzyme that catalyzes the first stage of lipid hydrolysis and is required for the metabolism of membrane lipids in plant cells. MeJA could improve the cold resistance of green bell pepper through the decreased expression and activity of the *PLD* gene [20]. The PLD activity in ‘Nanguo’ pears [7] was increased by core browning. Several environmental stressors such as cold, salt, and drought can affect the expression of *PLD* [43]. The application of MeJA to banana fruits resulted in an initial increase, followed by a decrease in the relative expression of *MaPLDγ*, *MaPLDα*, and *MaPLDζ*. This pattern may be attributed to the rapid activation of PLD signaling pathway in response to mechanical injury to banana fruits. Exogenous MeJA treatment significantly reduced the expressions of *MaPLDγ*, *MaPLDα*, and *MaPLDζ* in mechanically injured banana peels compared with those expressions in the control. Moreover, compared to the 50 μM MeJA group, the expressions of *MaPLDγ*, *MaPLDα*, and *MaPLDζ* were markedly decreased in the 10 μM MeJA group. It was shown previously that the application of MeJA prevented an increase in the PLD content and related gene expression, thereby protecting the integrity of cell membrane [10,20]. MeJA treatment may reduce the accumulation of PLD and PA by regulating *PLD* gene expression, as well as delay the aging process associated with membrane lipid degradation, which could improve the stress resistance of banana fruits and thus preserve the quality of the fruit during storage.

## 5. Conclusions

Mechanical damage has a significant impact on cell membrane permeability and the MDA content of fruits. This results in lipid peroxidation and electrolyte leakage, both of which accelerate ripening and aging processes of fruits. Our results demonstrated that 10 μM MeJA content in banana peels had the most significant effect. The analysis on enzymes and their components associated with membrane lipid metabolism further supported the significant effect of MeJA in maintaining the quality of postharvest banana fruits. MeJA levels were not only reflected in the inhibition of membrane lipid metabolic enzyme activities but also in reducing phospholipid degradation, which improved the tolerance to mechanical damage and repair capacity. This further supports the potential of MeJA as a plant defense signaling molecule and provides new insights into improving postharvest preservation of fruits.

From an economic perspective, the application of MeJA holds significant potential. MeJA can extend the shelf life of fruits and decrease economic losses due to spoilage and damage. Additionally, MeJA treatment may reduce the reliance on expensive chemical preservatives, lowering production costs. However, the optimal MeJA concentrations, the long-term effects of MeJA treatment, and the application methods for different fruit varieties, as well as potential trade-offs between MeJA treatment and other storage strategies, still need further investigation.

## Data Availability

The original contributions presented in the study are included in the article, further inquiries can be directed to the corresponding author.

## References

[1] Kuyu C., Tola Y. (2018). Assessment of banana fruit handling practices and associated fungal pathogens in Jimma town market, southwest Ethiopia. Food Sci. Nutr..

[2] Mai A.-D., Pankaj B.P., Rashid A.-Y., Hemanatha J., Zahir A.-A. (2023). Postharvest quality, technologies, and strategies to reduce losses along the supply chain of banana: A review. Trends Food Sci. Technol..

[3] Wu Y., Yang Q., Xie L., Yin C., Qu H., He J., Jiang Y., Li T. (2023). Possible mechanism of contribution of a secreted aspartic proteinase FpOPSB to the virulence of *Fusarium proliferatum* causing banana crown rot. Food Front..

[4] Xia Y., Lai Z., Do Y., Huang P. (2023). Characterization of microRNAs and gene expression in ACC oxidase RNA interference-based transgenic bananas. Plants.

[5] Li L., He X., Sun J., Li C., Ling D., Sheng J., You X., Li J., Liu G., Zheng F. (2017). Cloning characterization and functional expression of phospholipase Dα cDNA from banana (*Musa acuminate* L.). J. Food Qual..

[6] Li L., He X., Sun J., Li C., Ling D., Sheng J., Zheng F., Liu G., Li J., Tang Y. (2017). Responses of phospholipase D and antioxidant system to mechanical wounding in postharvest banana fruits. J. Food Qual..

[7] Sun Y., Sun H., Luo M., Zhou X., Zhou Q., Wei B., Cheng S., Ji S. (2020). Membrane lipid metabolism in relation to core browning during ambient storage of ‘Nanguo’ pears. Postharvest Biol. Technol..

[8] Chen Y., Sun J., Lin H., Lin M., Lin Y., Wang H., Hung Y. (2020). Salicylic acid treatment suppresses *Phomopsis longanae* Chi-induced disease development of postharvest longan fruit by modulating membrane lipid metabolism. Postharvest Biol. Technol..

[9] Li L., Yi P., Huang F., Tang J., Sun J., Duan X., Li J., Su Z., Ling D., Tang Y. (2022). Effects of phospholipase D inhibitors treatment on membrane lipid metabolism of postharvest banana fruit in response to mechanical wounding stress. Horticulturae.

[10] Peng Y., Mao L. (2011). Salicylic acid, ethephon, and methyl jasmonate induce the expression of phospholipase D in mechanically-wounded cucumber. J. Hortic. Sci. Biotechnol..

[11] Rojo E., Titarenko E., León J., Berger S., Vancanneyt G., Sánchez-Serrano J.J. (1998). Reversible protein phosphorylation regulates jasmonic acid-dependent and -independent wound signal transduction pathways in *Arabidopsis thaliana*. Plant J..

[12] Balusamy S.R.D., Rahimi S., Sukweenadhi J., Kim Y., Yang D. (2015). Exogenous methyl jasmonate prevents necrosis caused by mechanical wounding and increases terpenoid biosynthesis in *Panax ginseng*. Plant Cell Tissue Organ Cult..

[13] Wang S., Shi X., Liu F., Laborda P. (2021). Effects of exogenous methyl jasmonate on quality and preservation of postharvest fruits: A review. Food Chem..

[14] Reyes-Díaz M., Lobos T., Cardemil L., Nunes-Nesi A., Retamales J., Jaakola L., Alberdi M., Ribera-Fonseca A. (2016). Methyl jasmonate: An alternative for improving the quality and health properties of fresh fruits. Molecules.

[15] Liu Y., Liu Y., Chen Q., Yin F., Song M., Cai W., Shuai L. (2023). Methyl jasmonate treatment alleviates chilling injury and improves antioxidant system of okra pod during cold storage. Food Sci. Nutr..

[16] Huang T., Liu G., Zhu L., Liu J., Xiang Y., Xu X., Zhang Z. (2024). Mitigation of chilling injury in mango fruit by methyl jasmonate is associated with regulation of antioxidant capacity and energy homeostasis. Postharvest Biol. Technol..

[17] García-Pastor M.E., María S., Fabián G., Salvador C., Domingo M., Daniel V., Zapata P.J. (2019). Methyl jasmonate effects on table grape ripening, vine yield, berry quality and bioactive compounds depend on applied concentration. Sci. Hortic..

[18] Guan Y., Hu W., Jiang A., Xu Y., Sa R., Feng K., Zhao M., Yu J., Ji Y., Hou M. (2019). Effect of methyl jasmonate on phenolic accumulation in wounded broccoli. Molecules.

[19] Yu X., Zhang W., Zhang Y., Zhang X., Lang D., Zhang X. (2019). The roles of methyl jasmonate to stress in plants. Funct. Plant Biol..

[20] Ma M., Zhu Z., Cheng S., Zhou Q., Zhou X., Kong X., Hu M., Yin X., Wei B., Ji S. (2020). Methyl jasmonate alleviates chilling injury by regulating membrane lipid composition in green bell pepper. Sci. Hortic..

[21] Shuai L., Xue P., Liao L., Liu Y., Song M., Shang F., Cai W., Yin F., Cai J. (2024). Methyl jasmonate suppressed the pericarp browning in postharvest longan fruit by modulating membrane lipid and energy metabolisms. Postharvest Biol. Technol..

[22] Li T., Zhang J., Zhu H., Qu H., You S., Duan X., Jiang Y. (2016). Proteomic analysis of differentially expressed proteins involved in peel senescence in harvested mandarin fruit. Front. Plant Sci..

[23] Lin Y., Lin H., Zhang S., Chen Y., Chen M., Lin Y. (2014). The role of active oxygen metabolism in hydrogen peroxide-induced pericarp browning of harvested longan fruit. Postharvest Biol. Technol..

[24] Chen Y., Yu J., Lin H., Lin M., Lin Y., Zheng Y., Lin Y.F. (2022). Membrane lipids metabolism participates in the pulp breakdown of fresh longan caused by *Phomopsis longanae* Chi. Postharvest Biol. Technol..

[25] Yi P., Li L., Sun J., He X., Li C., Sheng J., Xin M., Ling D., Li Z., Tang Y. (2022). Characterization and expression of phospholipase D putatively involved in *Colletotrichum musae* disease development of postharvest banana fruit. Horticulturae.

[26] Kollárová R., Holková I., Rauová D., Bálintová B., Mikuš P., Obložinský M. (2017). HPLC analysis and biochemical characterization of LOX from *Eschscholtzia californica* Cham. Molecules.

[27] David N.B., Carlos P., Meltem S., Karen R. (2009). Phosphatidate degradation: Phosphatidate phosphatases (lipins) and lipid phosphate phosphatases. BBA Mol. Cell Biol. Lipids.

[28] Kue Foka I.C., Ketehouli T., Zhou Y., Li X.-W., Wang F., Li H. (2020). The emerging roles of diacylglycerol kinase (DGK) in plant stress tolerance, growth, and development. Agron. J..

[29] Shuai L., Li L., Sun J., Liao L., Duan Z., Li C., He X. (2020). Role of phospholipase C in banana in response to anthracnose infection. Food Sci. Nutr..

[30] Hong Y., Zhao J., Guo L., Kim S., Deng X., Wang G., Zhang G., Li M., Wang X. (2016). Plant phospholipases D and C and their diverse functions in stress responses. Prog. Lipid. Res..

[31] Dek M., Padmanabhan P., Subramanian J., Paliyath G. (2018). Inhibition of tomato fruit ripening by 1-MCP, wortmannin and hexanal is associated with a decrease in transcript levels of phospholipase D and other ripening related genes. Postharvest Biol. Technol..

[32] Zhang S., Lin Y., Lin H., Lin Y., Chen Y., Wang H., John S., Lin Y. (2018). *Lasiodiplodia theobromae* (Pat.) Griff. & Maubl.-induced disease development and pericarp browning of harvested longan fruit in association with membrane lipids metabolism. Food Chem..

[33] Fu X., Li F., Ali M., Song Y., Ding J., Kong X., Shang J., Zhao X., Li X., Zhang X. (2024). SlMsrB5-SlGRAS4 involved in methyl jasmonate-mediated ripening and quality of postharvest tomato fruit. Postharvest Biol. Technol..

[34] Han Y., Chen C., Yan Z., Li J., Wang Y. (2019). The methyl jasmonate accelerates the strawberry fruits ripening process. Sci. Hortic..

[35] Wei J., Wen X., Tang L. (2017). Effect of methyl jasmonic acid on peach fruit ripening progress. Sci. Hortic..

[36] Bagheri M., Esna-Ashari M. (2022). Effects of postharvest methyl jasmonate treatment on persimmon quality during cold storage. Sci. Hortic..

[37] Zhu L., Yu H., Dai X., Yu M., Yu Z. (2022). Effect of methyl jasmonate on the quality and antioxidant capacity by modulating ascorbate-glutathione cycle in peach fruit. Sci. Hortic..

[38] Liu Q., Xie H., Chen Y., Lin M., Hung Y., Lin H. (2022). Alleviation of pulp breakdown in harvested longan fruit by acidic electrolyzed water in relation to membrane lipid metabolism. Sci. Hortic..

[39] Glowacz M., Bill M., Tinyane P., Sivakumar D. (2017). Maintaining postharvest quality of cold stored ‘Hass’ avocados by altering the fatty acids content and composition with the use of natural volatile compounds—Methyl jasmonate and methyl salicylate. J. Sci. Food Agric..

[40] Song C., Wang K., Xiao X., Liu Q., Yang M., Li X., Feng Y., Li S., Shi L., Chen W. (2022). Membrane lipid metabolism influences chilling injury during cold storage of peach fruit. Food Res. Int..

[41] Wang C., Chen L., Peng C., Shang X., Lv X., Sun J., Li C., Wei L., Liu X. (2020). Postharvest benzothiazole treatment enhances healing in mechanically damaged sweet potato by activating the phenylpropanoid metabolism. J. Sci. Food Agric..

[42] Ma L., Zheng Y., Sang Z., Ge Y., Bai C., Fu A., Zuo J. (2023). Multi-omics analysis reveals the mechanism of calcium-reduced quality deterioration in mechanically injured green pepper fruit. Postharvest Biol. Technol..

[43] Guo X., Zhu W., Wang F., Wang H. (2024). Genome-wide investigation of the PLD gene family in tomato: Identification, analysis, and expression. Genes.

